# Development of a prognostic model for major postoperative complications in gastric cancer patients undergoing laparoscopic gastrectomy

**DOI:** 10.3389/fsurg.2026.1819934

**Published:** 2026-09-24

**Authors:** Huaying Liu, Hui Li, Fangfang Yang, Huiping Liang, Yun Qian, Hongtao Liu, Yu Shen, Xiaolong Ge

**Affiliations:** 1 Department of Medicine, Guangxi Health Science College, Guangxi, China; 2 Department of Vascular Surgery, Chinese PLA General Hospital, Beijing, China; 3 Department of Basic Medical Sciences, Guangxi Health Science College, Guangxi, China; 4 Department of General Surgery, School of Medicine, Sir Run Run Shaw Hospital, Zhejiang University, Hangzhou, Zhejiang, China; 5 Department of Nutrition and Food Safety, Zhejiang Provincial Center for Disease Control and Prevention, Hangzhou, Zhejiang, China

**Keywords:** gastric cancer, laparoscopic gastrectomy, nomogram, postoperative complications, skeletal muscle index

## Abstract

**Background:**

Early screening for gastric cancer (GC) patients undergoing laparoscopic gastrectomy (LG) at high risk for major postoperative complications is necessary for effective interventions.

**Methods:**

In this study, univariate analysis and LASSO regularized logistic regression were performed to screen predictors of major postoperative complications, and a predictive nomogram was established. The performance of the nomogram was validated with receiver operating curves, calibration curves, decision curve analysis and SHapley Additive exPlanations (SHAP).

**Results:**

In this study, a total of 410 GC patients undergoing LG were included, of whom 48 patients (11.71%) developed major postoperative complications. Multivariate analysis showed that age [Odds Ratio (OR) = 1.043], gender (OR = 0.328) and preoperative skeletal muscle index (SMI) (OR = 0.848) were independent risk factors for major postoperative complications. These variables were included in the nomogram. The concordance index of the model was 0.855 in the training cohort and 0.919 in the temporal validation. Among all features, SMI exhibited the highest global average absolute SHAP value.

**Conclusion:**

This individualized prognostic nomogram, established based on age, gender and SMI to predict the risk of major postoperative complications in GC patients undergoing LG, exhibited satisfactory predictive performance for major postoperative complications. The selected predictors were readily accessible in routine clinical practice and could be used as a reference for clinical decision-making. SMI showed superior predictive performance compared with conventional BMI, and emerged as the most powerful preoperative predictive indicator in this model.

## 1 Introduction

Gastric cancer (GC) is a common malignant tumour of the digestive system. Globally, an estimated 1.08 million new GC cases and more than 0.7 million GC deaths occurred in 2020 (1, 2). At present, tumour resection is still the main treatment for GC. Laparoscopic gastrectomy (LG) was first reported by Kitano in 1994 (3). Since then, LG has gradually been recognized by surgeons worldwide. Some scholars believe that LG can not only achieve the same effect of the traditional open surgery, but also benefit many patients with the advantages of minor trauma and accelerated recovery. A study from Lian et al. showed that patients who underwent LG for GC had a lower incidence of postoperative complications than those who underwent open gastrectomies (4). However, the incidence of postoperative complications after LG for GC has been persistently high (5). Many studies have shown that postoperative complications will prolong the length of hospital stay, increase hospitalization costs, increase mortality, and even affect the survival rate and survival of patients (6).

The known risk factors for postoperative complications include poor preoperative nutritional status, inflammatory indicators, obesity, older age, surgical methods, etc (7). However, these previous studies on complications after LG for GC have been limited by insufficient predictors (8–10). In addition, there is still no individualized prognostic nomogram for predicting the risk of major postoperative complications in patients undergoing LG for GC of all ages. Early identification of high-risk individuals via predictive modeling serves as a critical bridge to optimizing perioperative management and enabling personalized curative treatment for gastric cancer (11). Therefore, an effective and reliable risk prediction tool for major postoperative complications after LG for all-age GC is needed for the identification of the population at high risk for postoperative complications after LG, allowing for corresponding measures to prevent the occurrence of major postoperative complications in this population. The present study had the inclusion of skeletal muscle index (SMI) and covered a broader age range which aimed at investigating the risk factors for major postoperative complications. By analysing the clinical data of GC patients who underwent LG in our hospital to develop an individualized prognostic nomogram and provide a strategy for the risk stratification of the patients with GC.

## 2 Materials and methods

### 2.1 Patients

Our study used the data of GC patients who underwent LG from January 2016 to December 2025 in the Sir Run Run Shaw Hospital, to establish a prognostic model. Patients in the internal validation cohort were recruited from Sir Run Run Shaw Hospital between January 2016 and March 2023. For the temporal validation cohort, participants were enrolled from Sir Run Run Shaw Hospital from March 2023 to December 2025. The inclusion criteria were as follows: (1) definitively diagnosed GC according to the pathological diagnosis, (2) underwent LG, (3) voluntary acceptance of enhanced recovery after surgery (ERAS). The core measures of ERAS implemented in this study included: (1) abandonment of traditional preoperative fasting; (2) avoidance of routine preoperative bowel preparation (e.g., oral laxatives); (3) encouragement of early ambulation within 24 h postoperatively; and (4) early removal of the urinary catheter (usually within 24–48 h after surgery). The exclusion criteria were as follows: (1) suffered from other cancer simultaneously, (2) LG was only for palliative management, (3) distant metastasis, (4) incomplete data including unqualified CT images, and (5) severe kidney dysfunction (estimated glomerular filtration rate < 30 mL/min/1.73m^2^). The study was approved by the Ethics Committee of Sir Run Run Shaw Hospital and GuangXi Health Science College. For minor participants under 18 years of age, written informed consent was obtained from their legal guardians or parents in accordance with the Declaration of Helsinki.

### 2.2 Clinical data collection

Patient data, including baseline characteristics and laboratory examination results from the hospital database, were all collected. Based on the variables analysed in previous studies on risk factors for postoperative complications after LG for GC (8–11), we chose gender, age, comorbidities, SMI and body mass index (BMI) as the analysed baseline characteristics. The preoperative laboratory examination results were obtained in a week before surgery, including red blood cells (RBCs), white blood cells (WBCs), lymphocytes, albumin (ALB), haemoglobin (Hb), platelets (PLTs), C-reactive protein (CRP), Carbohydrate antigen 199, and Carcinoembryonic antigen (CEA). The surgical data included type of operative procedure, operation time and intraoperative blood loss. Postoperative complications were assessed according to the Clavien-Dindo classification (12). During the patients’ postoperative hospital stay, Clavien-Dindo grade I or II complications were defined as mild, and grade III–IV complications were defined as major. Patients were categorized into the major postoperative complication group and the No/Mild postoperative complication group.

### 2.3 Statistical analysis

This study was designed as a single-center retrospective study. In this study, Social Sciences (SPSS) 22.0 and R software (version 4.2.1 based on the Windows 11 64-bit operating system) was used for statistical analysis. Continuous variables were first tested for normality using a Quantile-Quantile Plot. Normally distributed continuous variables were described by the mean ± standard deviation (SD) and Student's t test was used for comparisons between groups. Skewed measurement data were expressed by the median (Percentiles 25, Percentiles 75), and Mann–Whitney U test was used for statistical analysis. Patients with incomplete data were excluded. Categorical variables were presented as numbers (percentages), and the Pearson *χ*^2^ test or Fisher's exact test was used for comparisons between groups. Univariate analysis and Least Absolute Shrinkage and Selection Operator (LASSO) was performed, and the selected variables from LASSO were included in the multivariate analysis. Next, R software was used to establish the nomogram with the predictive factors. The performance of the nomogram was validated with receiver operating curves (ROCs), calibration curves, decision curve analysis (DCA) and SHapley Additive exPlanations (SHAP). *P* < 0.05 was considered statistically significant. Sample size considerations were based on the events-per-variable (EPV) rule for logistic regression models. The total number of events (patients with complications) in the study was 48, yielding an EPV of 16 for the three predictors included in the final model. This EPV value meets the recommended threshold of ≥10, ensuring adequate statistical power for stable coefficient estimation and reducing the risk of overfitting.

## 3 Results

### 3.1 Incidence of major postoperative complications in GC patients underwent LG

All potential study subjects underwent rigorous eligibility screening in accordance with the predefined inclusion and exclusion criteria. Data from subjects who failed to meet the criteria were excluded from the analysis. Finally, a total of 410 medical records that fully complied with the inclusion criteria were enrolled in this study, of whom 297 (72.44%) were male. Our cohort consisted of patients undergoing laparoscopic distal gastrectomy (LDG) and laparoscopic total gastrectomy (LTG). The distribution of these two surgical subtypes is summarized in Table 1. Forty-eight patients (11.71%) developed major postoperative complications, while 362 patients (88.29%) had No/Mild postoperative complications, as shown in Table 1. The most common first major postoperative complications were pleural effusion and intra-abdominal hemorrhage, each occurring in 8 cases (16.67%, 8/48), as shown in Table 2. The number of outcome events in both the training and temporal validation cohorts satisfied the 10 events per variable standard, ensuring stable model construction and avoiding overfitting.

**Table 1:** Univariate analyses of predictive factors associated with major postoperative complications in gastric cancer.

Characteristic	Major postoperative complications (*n* = 48)	No/Mild postoperative complications (*n* = 362)	P	95% CI of Univariate Odds Ratios
Age, years	67.8 ± 8.9	63.0 ± 11.3	0.005	1.013–1.076
Male, male	39 (81.25)	258 (71.27)	0.146	0.280–1.300
BMI, kg/m^2^	21.2 ± 2.3	22.6 ± 3.0	0.003	0.753–0.945
Diabetes mellitus, n(%)	3 (6.25)	49 (13.54)	0.154	0.778–8.960
Hypertension, n(%)	24 (50)	140 (38.67)	0.132	0.320–1.092
Previous abdominal surgery, n(%)	8 (16.67)	86 (23.76)	0.272	0.639–3.210
Preoperative hemoglobin, g/L	126.0 ± 26.5	122.4 ± 24.2	0.343	0.993–1.019
Preoperative CRP, mg/L	5.2 ± 10.2	5.2 ± 11.5	0.990	0.974–1.027
Preoperative WBC,×10^9^/L	6.0 ± 1.6	5.8 ± 1.7	0.367	0.912–1.283
Preoperative RBC, ×10^12^/L	4.10 ± 0.79	4.12 ± 0.69	0.839	0.625–1.464
Preoperative platelets, ×10^9^/L	223.4 ± 59.5	219.9 ± 74.7	0.755	0.997–1.005
Preoperative albumin, g/L	39.2 ± 5.3	39.0 ± 5.0	0.802	0.946–1.069
Preoperative total lymphocytes,×10^12^/L	1.49 ± 0.59	1.54 ± 0.64	0.607	0.534–1.443
Preoperative SMI, cm^2^/m^2^	42.5 ± 10.2	56.2 ± 8.8	<0.001	0.824–0.893
CA199, U/mL	25.0 ± 37.1	25.5 ± 71.1	0.963	0.996–1.005
CEA, μg/L	7.2 ± 15.3	6.0 ± 25.8	0.735	0.992–1.012
Types of operative procedure, n(%)			0.629	0.582–2.057
Distal gastrectomy	30 (62.5)	239 (66.02)	-	
Total gastrectomy	18 (37.5)	123 (33.98)	-	
Estimated blood loss, mL	103.1 ± 59.9	99.2 ± 122.4	0.826	0.998–1.003

SMI, skeletal muscle index; BMI, body mass index; CA19-9, Carbohydrate antigen 199; CEA, carcinoembryonic antigen, 95% CI = 95% confidence interval. The column “95% CI of Univariate Odds Ratios” only presents the confidence interval range corresponding to univariate odds ratios.

**Table 2:** Statistics on the first major complication in patients.

Major postoperative complications	All (*n* = 48)	Major postoperative complications	All (*n* = 48)
Pleural effusion, n(%)	8 (16.67)	Postoperative gastroplegia syndrome, n(%)	2 (4.17)
Gastrointestinal bleeding, n(%)	3 (6.25)	Afferent loop obstruction, n(%)	1 (2.08)
Anastomotic stenosis, n(%)	3 (6.25)	Anastomotic leakage, n(%)	3 (6.25)
Septic shock, n(%)	1 (2.08)	Abdominopelvic collection, n(%)	13 (27.08)
Intra-abdominal bleeding, n(%)	8 (16.67)	Intra-abdominal abscess, n(%)	3 (6.25)
Postoperative bowel obstruction, n(%)	2 (4.17)	Intestinal perforation, n(%)	1 (2.08)

### 3.2 Univariate analysis of major postoperative complications

Patients were divided into major postoperative complication group and No/Mild postoperative complication group based on whether they had major postoperative complications. There were significant differences in age, BMI and SMI between the two groups (all *P* < 0.05), as shown in Table 1. To avoid overfitting and select optimal predictors, we performed LASSO penalized logistic regression with 10-fold cross-validation. The optimal *λ* value was determined based on minimizing the binomial deviance. The selected variables included age, gender, diabetes mellitus, hemoglobin level, and SMI. The LASSO coefficient profile and cross-validation curve are presented in Figure 1. Notably, this LASSO-derived candidate pool was only used to eliminate irrelevant predictors, and further clinical multivariate adjustment was conducted to determine the final predictors of the regression model.

**Figure 1:**
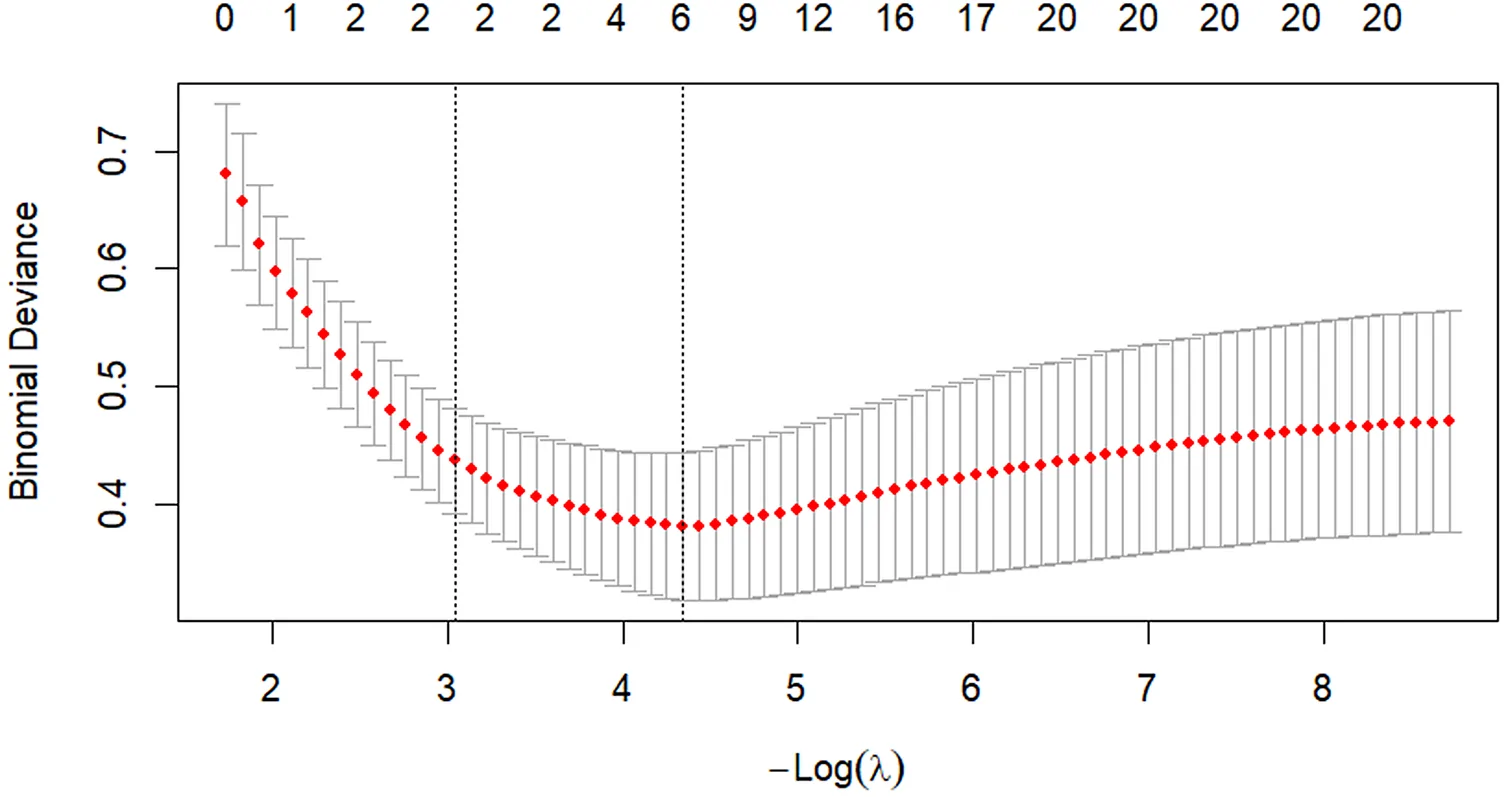
LASSO regression with 10-fold cross-validation for variable selection. Age = 0.00222695; BMI = 0.00000000; SMI = −0.09838349.

### 3.3 Multivariate analysis of major postoperative complications

Logistic regression analysis was performed to determine the independent risk factors for major postoperative complications. The selected variables from LASSO were used as independent variables, and the occurrence of major postoperative complications was used as the dependent variable. Additionally, BMI was penalized to zero and excluded during the initial LASSO screening step due to multicollinearity with SMI. Gender was retained in the LASSO preliminary candidate pool but exhibited non-significant univariate correlation with major postoperative complications. However, to eliminate confounding bias originating from inherent sex differences in skeletal muscle mass distribution, gender was mandatorily incorporated as an adjustment covariate in the revised final multivariate model, as shown in Table 3. The results showed that age [Odds Ratio (OR) = 1.043, 95% CI = 1.002–1.085, *P* = 0.041], gender (OR=0.328, 95% CI = 0.129–0.837, *P* = 0.020) and SMI (OR=0.848, 95%CI = 0.811–0.886, *P* < 0.001) were identified as independent risk factors for major postoperative complications (all *P* < 0.05) (Table 3). The variance inflation factor (VIF) was calculated to evaluate multicollinearity. All VIF values (age: 1.112, gender: 1.082, SMI: 1.017) were below 5, suggesting no problematic multicollinearity.

**Table 3:** Multivariate analyses of predictive factors associated with major postoperative complications in gastric cancer.

Characteristic	OR	95% CI	*P* value
Age	1.043	1.002–1.085	0.041
Preoperative Hb	1.011	0.996–1.027	0.148
Preoperative SMI	0.848	0.811–0.886	<0.001
Gender	0.328	0.129–0.837	0.020
DM	2.486	0.657–9.407	0.180

Variance inflation factor: age = 1.112, Hb = 1.123, SMI = 1.017, gender = 1.082, DM = 1.014.

SMI, skeletal muscle index; BMI, body mass index; Hb, hemoglobin.

### 3.4 Establishment and validation of a nomogram model for the risk of major postoperative complications

Based on the results of the multivariate analysis, the independent risk factors identified in multivariate analysis were introduced into R software to establish an individualized predictive nomogram risk prediction model, as shown in Figure 2. The ROC (Figure 3), calibration curve (Figure 4), DCA (Figure 5), and SHAP Summary Plot (Figure 6) in the training cohort were plotted. The C-index of the model was 0.855 in the training cohort. The calibration plot showed that the calibrated solid line was very close to the ideal line. For temporal validation, we retrospectively enrolled data from 205 GC patients who underwent LG. The incidence of major postoperative complications was 13.65% (28/205) in the temporal validation cohort, vs. 11.71% (48/410) in the training cohort. The performance of our developed nomogram in predicting major postoperative complications was satisfactory, with a C-index of 0.919. The comprehensive predictive performance of the nomogram, including sensitivity, specificity, accuracy and F1-score with 95% CI, was summarized in Table 4. Furthermore, analysis of the model's global feature importance using SHAP values revealed that, in predicting the risk of major postoperative complications in GC, the SMI had the highest global average absolute SHAP value.

**Figure 2:**
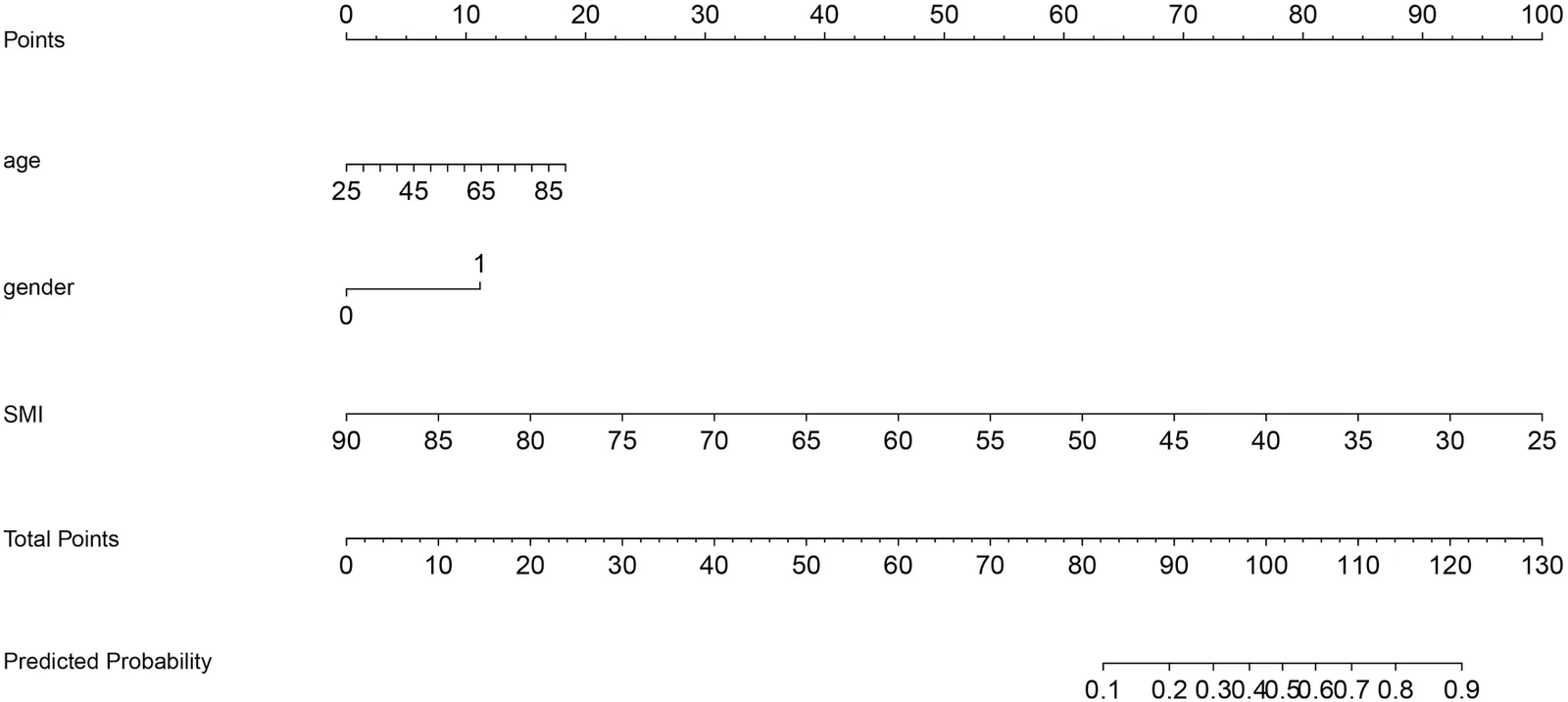
A nomogram predicting the risk of major postoperative complications in gastric cancer patients undergoing laparoscopic gastrectomy.

**Figure 3:**
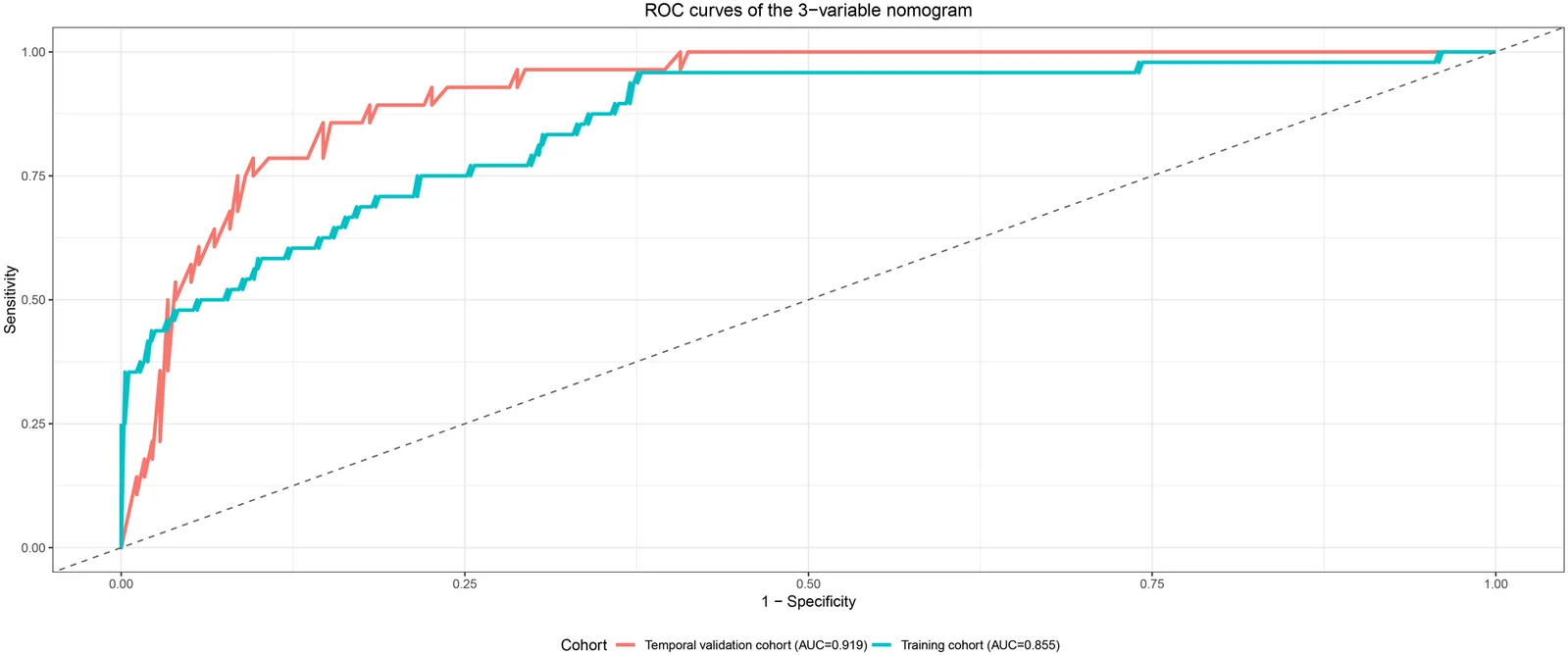
ROC of the nomogram in the training and temporal validation cohorts.

**Figure 4:**
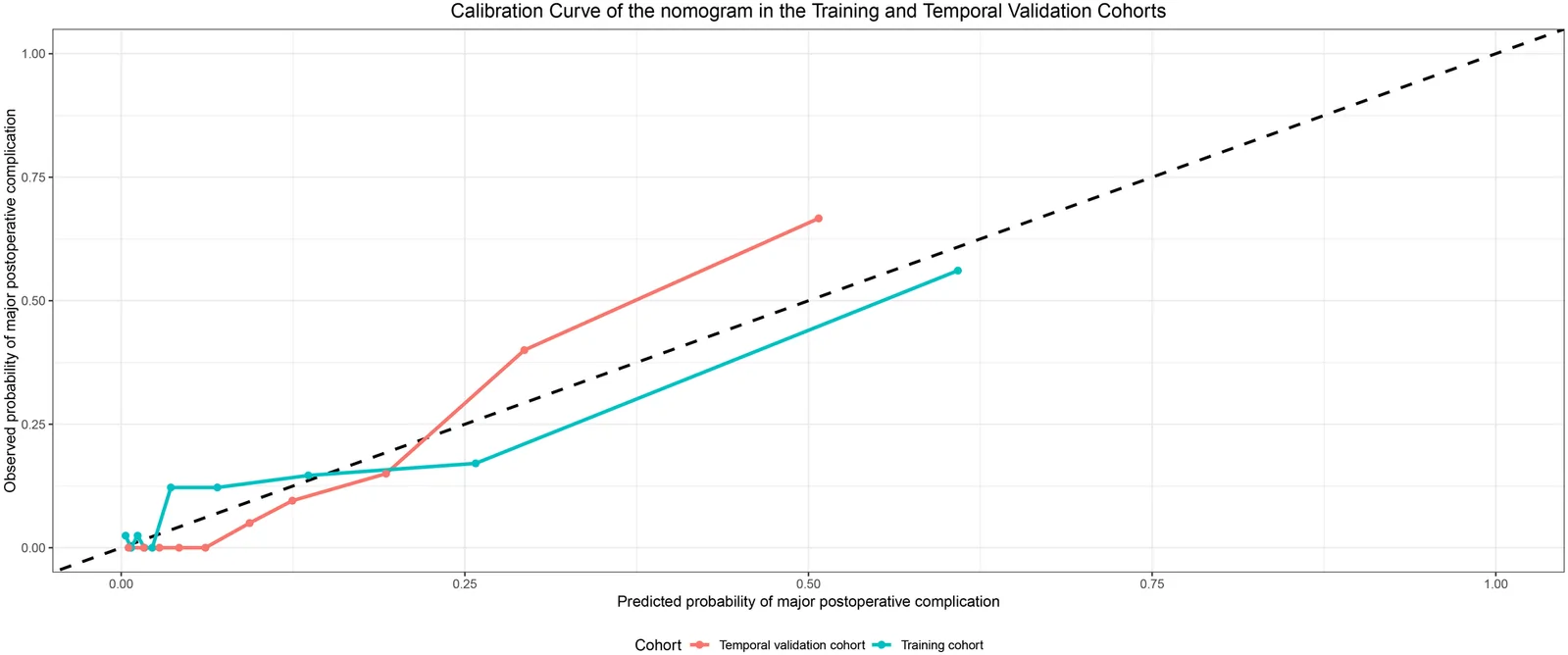
Calibration curve of the nomogram in the training and temporal validation cohorts. Left: Calibration Curve of the nomogram in the Training Validation Cohorts; Right: Calibration Curve of the nomogram in the Temporal Validation Cohorts.

**Figure 5:**
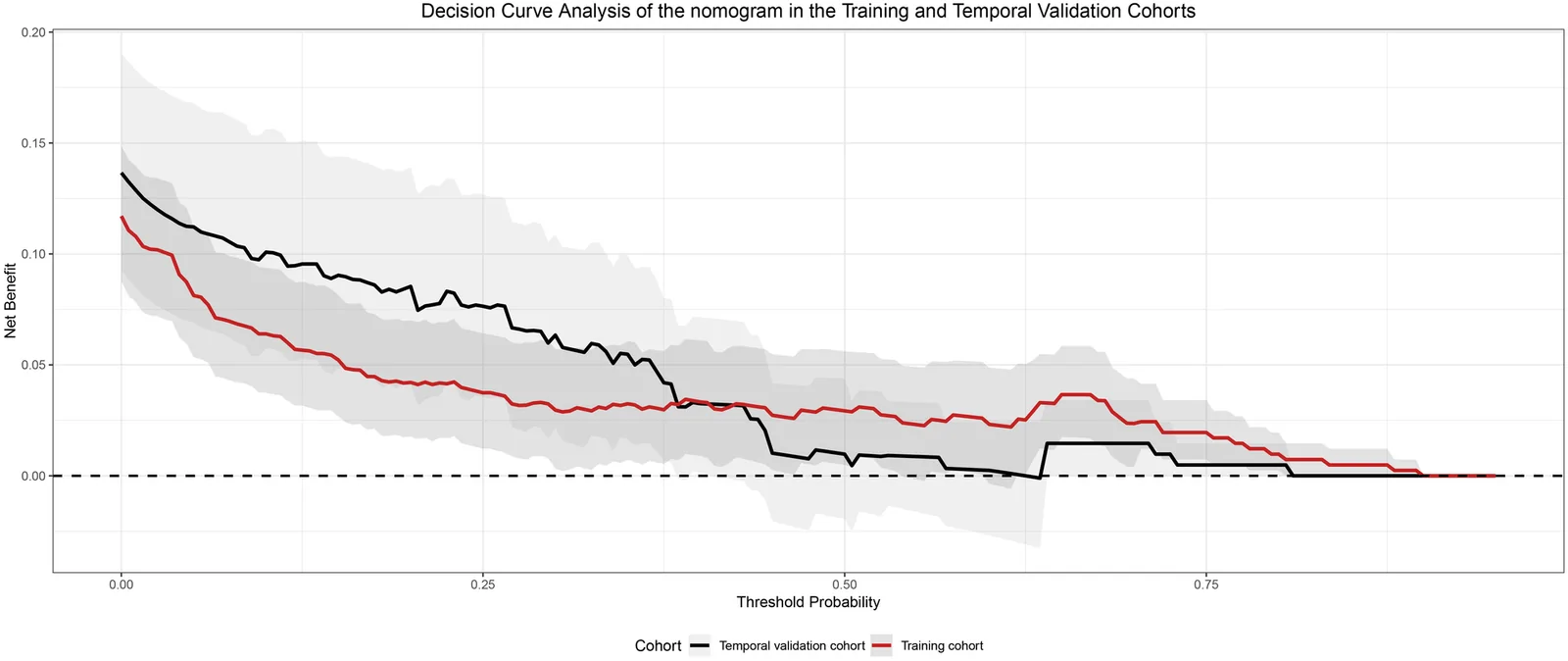
Decision curve analysis of the nomogram in the training and temporal validation cohorts. Left: Decision Curve Analysis of the nomogram in the Training Validation Cohorts; Right: Decision Curve Analysis of the nomogram in the Temporal Validation Cohorts.

**Figure 6:**
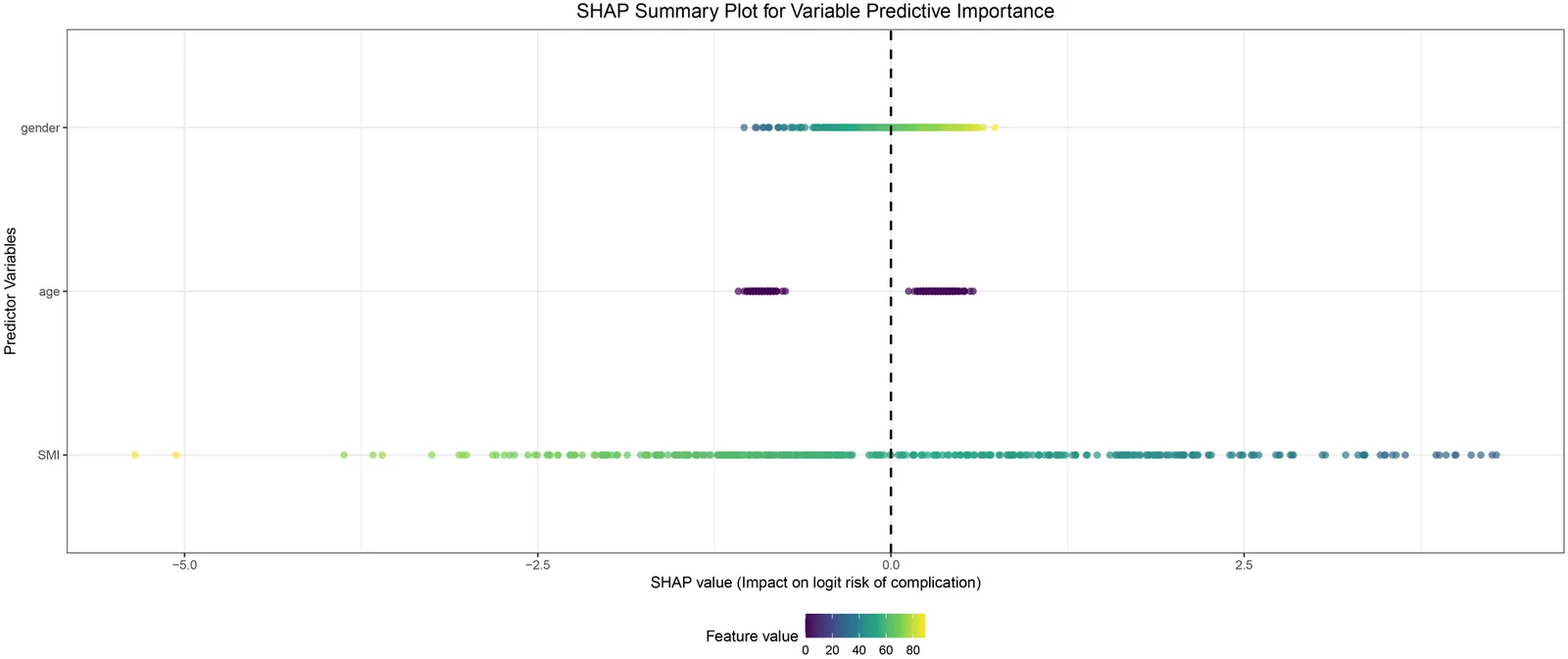
SHAP plots of the nomogram.

**Table 4:** Performance of the prediction model in the training and temporal validation cohorts.

Metric	Training Validation	Temporal Validation
Sensitivity (95% CI)	0.917 (0.804–0.967)	0.821 (0.631–0.939)
Specificity (95% CI)	0.663 (0.613–0.710)	0.503 (0.427–0.579)
Accuracy (95% CI)	0.693 (0.646–0.735)	0.507 (0.437–0.578)
Precision (95% CI)	0.265 (0.204–0.337)	0.667 (0.384–0.882)
F1 score	0.411	0.465

## 4 Discussion

This study developed an individualized predictive nomogram for major postoperative complications in GC patients after LG. This model is expected to be used for identification of high-risk groups for major postoperative complications in GC patients receiving LG; this allows surgeons to provide corresponding preventative measures and treatment to these patients in a timely manner, which is conducive to reducing the incidence of major postoperative complications, medical economic expenditure and burden to the patients and pressure on the medical staff for diagnosis, treatment and nursing. This model facilitates early risk stratification and targeted postoperative intervention, supporting the clinical rationale of model-guided perioperative management in gastric cancer.

The results showed that the incidence of major postoperative complications was 11.71%, which was consistent with the results reported by Huang et al, who developed a predictive scoring system based on the records of 2,170 GC patients that underwent LG (13). According to the independent risk factors for GC in multivariate analysis, the patients were divided into different risk groups, and the overall complication rates ranged from 8.3% to 29.9%. Additionally, the concept of ERAS was implemented in the present study, and these ERAS-related interventions might also be the potential factors contributing to the relatively low incidence of major postoperative complications, though this association requires further verification in larger cohorts (14, 15).

To the best of our knowledge, although similar models already exist, but there is no research has focused on establishing an individualized risk predictive model for major postoperative complications in GC patients undergoing LG of all ages, and the inclusion of SMI has added incremental value to the research. Tu et al. developed a risk scoring system consisting of three predictors including age ≥ 65 years, haemoglobin ≤ 8.0 g/dL and malnutrition to assess the incidence of and factors related to anastomotic leakage after open radical gastrectomy for GC, but their study did not focus on LG (16). Li et al. established a risk scoring system consisting of 3 predictors (age > 70 years, male sex, and BMI > 25 kg/m^2^) to evaluate the risk factors affecting unplanned reoperation after LG for GC (17). However, their study did not focus on postoperative complications (18). Chen et al. constructed a predictive model only focus on elderly GC patients after LG (19). To comprehensively explore the risk factors for major postoperative complications for all-age GC patients after LG and improve patient prognosis, we developed a prognostic model consisting of three variables (age, gender, and SMI), which were identified as independent risk factors for major postoperative complications.

This study demonstrated that continuous age was an independent risk factor for major postoperative complications, with increasing age correlating with elevated postoperative risk, consistent with prior reports (20, 21). The reasons may be as follows: As patients increase in age, their body function declines and multiple organ functions get weakening, their immune capacity and tolerance decreases to varying degrees, their general condition weakens, their postoperative compensatory and tissue repair abilities decline, their postoperative wound healing slows, their pulmonary function becomes significantly reduced, their blood viscosity becomes elevated, and their anti-infective ability weakens. Hence, various complications are likely to occur, resulting in an unfavorable outcome (22, 23). For elderly patients, once major complications occur after surgery, the mortality risk increases significantly (24). Therefore, we should pay more attention to elderly GC patients, and take necessary interventions to improve body function and reduce the occurrence of major postoperative complications.

In addition, our study identified female sex as a protective factor against major postoperative complications in gastric cancer patients receiving laparoscopic gastrectomy, which is consistent with the findings of previous studies (25, 26). A study by Tokunaga et al. analyzed clinical data from 988 GC patients who received minimally invasive distal gastrectomy. Their results demonstrated that male sex served as an independent risk factor for postoperative complications (26). Gender differences are closely linked to patients’ endocrine levels, physiological reserve and baseline inflammatory status. Compared with male counterparts, female patients typically accumulate less visceral adipose tissue and present a blunted systemic inflammatory response after minimally invasive abdominal surgery, alongside enhanced physiological stress tolerance during perioperative periods. Male GC patients are far more likely to have a history of chronic tobacco smoking and heavy alcohol consumption before surgery; meanwhile, pooled evidence from meta-analyses of GC cohorts confirms that male sex is an independent risk factor for preoperative sarcopenia (25, 27, 28). Therefore, targeted preoperative optimization strategies tailored to male patients, such as smoking cessation and nutritional support to preserve skeletal muscle mass, can strengthen perioperative immune function, improve surgical tolerance, reduce complication incidence and optimize short-term postoperative recovery. However, we also noted that gender showed no statistically significant intergroup difference in univariate analysis (*P* = 0.146), yet it was retained by LASSO regularization and verified as an independent protective predictor in multivariate logistic regression (OR = 0.328, 95%CI 0.129–0.837, *P* = 0.020). This discrepancy may be attributed to confounding effects of age and SMI that mask its crude between-group difference in univariate comparison, which highlights the necessity of multivariate adjustment to eliminate interference from coexisting risk factors.

Our study also demonstrated that a low SMI was an independent risk factor for major postoperative complications in GC patients. Although univariate analysis revealed significant intergroup differences in BMI, this variable was ultimately eliminated by LASSO regularization. Even when BMI was forcibly incorporated into multivariate logistic regression alongside age, gender and SMI, it failed to emerge as an independent risk factor for major postoperative complications. One potential explanation for the exclusion of BMI is its multicollinearity with SMI, leading to its removal by the LASSO model. Since SMI provides more precise information on muscle mass and nutritional status than conventional BMI, it was retained as a more representative predictor, whereas BMI was automatically penalized to zero. This selection indicated that SMI had superior independent predictive value for postoperative complications. Compared with BMI, which only reflects overall body weight status, SMI directly evaluates skeletal muscle mass, which is closely related to surgical tolerance, stress response, and postoperative recovery. This finding was consistent with several previous studies, which suggested that compared with BMI, SMI could be a more reliable tool for evaluating the nutritional status and disease severity of GC patients. It is accepted that malnutrition is an important risk factor for clinical outcomes in cancer disease. Several studies have also shown that preoperative sarcopenia, rather than BMI, is an independent risk factor for major postoperative complications in GC patients following radical gastrectomy (29, 30). Sun et al. also reported that sarcopenia served as an independent predictor of long-term survival in patients with a normal BMI. Additionally, they recommended sarcopenia for use in nutritional risk screening (31). The underlying reason may be that muscle mass in GC patients not only reflects nutritional status but is also directly associated with immune function and stress tolerance. Preoperative sarcopenia may reduce tissue repair efficiency and increase susceptibility to infection, thereby elevating the risk of complications. Therefore, although BMI performs well in assessing patients’ nutritional status, relying solely on BMI to evaluate nutritional risk may have limitations. Clinicians should not rely only on BMI for nutritional assessment; instead, paying attention to muscle reserve status (as reflected by SMI) is also of great importance.

The nomogram is a simple, graphical, predictive model that shows integrated effect of each included variable on the outcome of interest, provides a visualization of the multivariate analysis results, and is easy to promote in clinical practice (32). Our research also developed a prognostic model for major postoperative complications in all-age GC patients after LG. The three variables identified in our study were age, gender and SMI. Since these variables are all relatively easy to obtain clinically, our nomogram may be useful and applicable. The C-index for the training cohort was 0.855, a value indicative of strong discriminative ability, which aligned with the benchmark for well-performing prognostic models in clinical research. Temporal validation showed the performance of our developed nomogram in predicting major postoperative complications was satisfactory, with a C-index of 0.919. To further elucidate the predictive logic and interpretability of the nomogram, SHAP analysis was conducted on individual patient samples. SHAP summary plot (Figure 6) visualized the magnitude and direction of each variable's predictive effect. SMI showed the widest SHAP value distribution and ranked first in predictive importance, outperforming gender and age. Lower SMI significantly increased the logit risk of postoperative complications, whereas male sex and older age also contributed to elevated risk. The SHAP findings further validated the superior independent predictive value of SMI relative to conventional BMI. Collectively, these findings confirm that our constructed nomogram not only exhibits favorable predictive efficacy in temporal validation cohorts but also possesses clear interpretability, with SMI identified as the dominant factor influencing the prediction of major postoperative complications in GC patients undergoing LG. Calibration analysis (Figure 4) further supported the robust predictive performance of our anthropometric nomogram. Although mild directional bias existed for moderate and high-risk patients across training and temporal validation cohorts, the two curves maintained generally good concordance with the ideal reference line, especially for low-risk individuals. DCA of the nomogram also showed the training curve retained positive net benefit across the full 0–0.9 probability range, while the validation curve delivered favorable clinical returns for thresholds below 0.68. Such consistent net gain above the zero-intervention baseline proved that the model could effectively guide individualized preoperative risk stratification and targeted intervention in real-world practice, even when generalized to an independent temporally separated patient population. The narrow confidence intervals surrounding both curves also reflected stable predictive performance without excessive statistical uncertainty (33).

As shown in Table 4, our nomogram achieved high sensitivity in both cohorts, which enables effective screening of patients at high risk of major postoperative complications. However, its specificity and overall accuracy were suboptimal, especially in the temporal validation cohort, indicating limited performance when stratifying patients by a single fixed threshold. Of note, the threshold used for Table 4 was determined by maximizing the Youden index within the training cohort. This cut-off prioritizes high sensitivity to avoid missing patients with high complication risk, which consequently leads to compromised specificity and low overall accuracy in the temporal validation cohort. Despite this categorical classification limitation, the model maintains stable global predictive performance and clinical utility supported by favorable C-index, calibration curves and DCA results. Overall, in contrast to previous studies on risk factors for postoperative complications after LG for GC patients, our study included patients’ baseline characteristics and perioperative laboratory examination results, such as SMI to establish the prognostic nomogram model. The clinical data needed for the model are easy to obtain, allowing the model to provide a reference for optimizing postoperative management and preventing and treating major postoperative complications. After further development of applications or scoring systems, this model has potential application value in clinical practice.

## 5 Limitation

Nonetheless, our study also has some limitations. Firstly, our nomogram was designed only to predict the prediction of short-term complications, which lack of follow-up analysis of long-term prognosis. Secondly, our study is a single-center retrospective and complete case analysis with unavoidable biases. Regarding sample size, we should have referenced models in previous studies to determine the sample size, but there still exists a lack of research about developing an individualized prognostic nomogram for predicting the risk of major postoperative complications in GC patients undergoing LG. Thirdly, conducting multi center external validation can improve the extrapolation of the model. Fourth, given that the current sample size is still insufficiently large, the continuous conduct of subgroup analyses in future research—such as stratification by gastrectomy type, tumor stage, and ERAS compliance—will help enhance the robustness of the research findings. Fifth, resection type was not significantly associated with major postoperative complications in univariable analysis and was excluded from the multivariable model. However, we did not separately assess the discriminative performance of the final model within LDG and LTG subgroups, so subtype-specific predictive performance needs further validation in future studies. Finally, the threshold derived from maximizing the Youden index was optimized to maintain high sensitivity for screening, which consequently leads to suboptimal specificity and overall accuracy in the temporal validation cohort. Clinicians should avoid rigid binary risk stratification using this single cut-off, as the nomogram is designed to estimate continuous individual risk probabilities rather than deliver dichotomized clinical decisions.

## 6 Conclusion

Age, gender and SMI are independent risk factors for major postoperative complications in all-age GC patients undergoing LG. The predictive nomogram exhibits satisfactory predictive performance for major postoperative complications in all-age GC patients after LG. After future multi center and large-scale validation, this model may be useful for identifying high-risk GC patients and conducive to implementing preventive and therapeutic measures.

## Data Availability

The original contributions presented in the study are included in the article/Supplementary Material, further inquiries can be directed to the corresponding author/s.
